# Biomarker profiles of coagulopathy and alveolar epithelial injury in acute respiratory distress syndrome with idiopathic/immune-related disease or common direct risk factors

**DOI:** 10.1186/s13054-019-2559-6

**Published:** 2019-08-19

**Authors:** Kansuke Koyama, Shinshu Katayama, Ken Tonai, Jun Shima, Toshitaka Koinuma, Shin Nunomiya

**Affiliations:** Division of Intensive Care, Department of Anesthesiology & Intensive Care Medicine, Jichi Medical University School of Medicine, 3311-1 Yakushiji, Shimotsuke, Tochigi 329-0498 Japan

**Keywords:** Acute respiratory distress syndrome, Common risk factors, Subtypes, Coagulopathy, Alveolar epithelial injury

## Abstract

**Background:**

Altered coagulation and alveolar injury are the hallmarks of acute respiratory distress syndrome (ARDS). However, whether the biomarkers that reflect pathophysiology differ depending on the etiology of ARDS has not been examined. This study aimed to investigate the biomarker profiles of coagulopathy and alveolar epithelial injury in two subtypes of ARDS: patients with direct common risk factors (dARDS) and those with idiopathic or immune-related diseases (iARDS), which are classified as “ARDS without common risk factors” based on the Berlin definition.

**Methods:**

This retrospective, observational study included adult patients who were admitted to the intensive care unit (ICU) at a university hospital with a diagnosis of ARDS with no indirect risk factors. Plasma biomarkers (thrombin–antithrombin complex [TAT], plasminogen activator inhibitor [PAI]-1, protein C [PC] activity, procalcitonin [PCT], surfactant protein [SP]-D, and KL-6) were routinely measured during the first 5 days of the patient’s ICU stay.

**Results:**

Among 138 eligible patients with ARDS, 51 were excluded based on the exclusion criteria (*n* = 41) or other causes of ARDS (*n* = 10). Of the remaining 87 patients, 56 were identified as having dARDS and 31 as having iARDS. Among the iARDS patients, TAT (marker of thrombin generation) and PAI-1 (marker of inhibited fibrinolysis) were increased, and PC activity was above normal. In contrast, PC activity was significantly decreased, and TAT or PAI-1 was present at much higher levels in dARDS compared with iARDS patients. Significant differences were also observed in PCT, SP-D, and KL-6 between patients with dARDS and iARDS. The receiver operating characteristic (ROC) analysis showed that areas under the ROC curve for PC activity, PAI-1, PCT, SP-D, and KL-6 were similarly high for distinguishing between dARDS and iARDS (PC 0.86, *P* = 0.33; PAI-1 0.89, *P* = 0.95; PCT 0.89, *P* = 0.66; and SP-D 0.88, *P* = 0.16 vs. KL-6 0.90, respectively).

**Conclusions:**

Coagulopathy and alveolar epithelial injury were observed in both patients with dARDS and with iARDS. However, their biomarker profiles were significantly different between the two groups. The different patterns of PAI-1, PC activity, SP-D, and KL-6 may help in differentiating between these ARDS subtypes.

**Electronic supplementary material:**

The online version of this article (10.1186/s13054-019-2559-6) contains supplementary material, which is available to authorized users.

## Background

Acute respiratory distress syndrome (ARDS) comprises acute-onset respiratory failure, which is characterized by hypoxemia and radiographic bilateral lung opacities that result from various direct or indirect injuries to the pulmonary parenchyma or vasculature [1]. The most recent Berlin definition provides common risk factors for ARDS, which are classified as direct factors (e.g., pneumonia, aspiration of gastric contents) or indirect factors (e.g., non-pulmonary sepsis, major trauma, pancreatitis) [2]. Some patients presenting with ARDS, however, lack exposure to common risk factors, resulting in the condition called an ARDS “imitator” or “mimic” [3, 4]. In a large cohort study, Gibelin et al. reported a 7.5% prevalence of ARDS without a common risk factor [5]. A secondary analysis of the LUNG SAFE study confirmed that 8.3% of ARDS patients had no common risk factors that were identified when ARDS was recognized [6].

These ARDS patients who lacked exposure to common risk factors can be categorized as having immune, idiopathic, drug-induced, and malignant diseases [6, 7]. Connective tissue disease-associated interstitial lung disease (CTD-ILD) is considered to be a main cause of immune-related forms of ARDS. CTD-ILD may precede the clinical and laboratory manifestations of CTD and therefore could present as lone ARDS [8]. Acute onset or acute exacerbation of idiopathic interstitial lung diseases may refer to idiopathic forms of ARDS. Although no risk factors or causes are identified in this subgroup of ARDS, recent studies have shown that many patients with idiopathic interstitial pneumonia have clinical features that suggest an underlying immune process, indicating that the pathobiology of idiopathic and immune-related diseases may partially overlap [9, 10]. Early identification of these subsets of ARDS based on the pathophysiology is of clinical interest and may lead to the development of specific therapeutic intervention. However, the lesions of these idiopathic and immune-related ARDS may be mostly limited to the lung, and it is often difficult in the acute phase to distinguish between idiopathic/immune-related diseases and ARDS with common direct risk factors, based solely on the clinical findings.

**Fig. 1:**
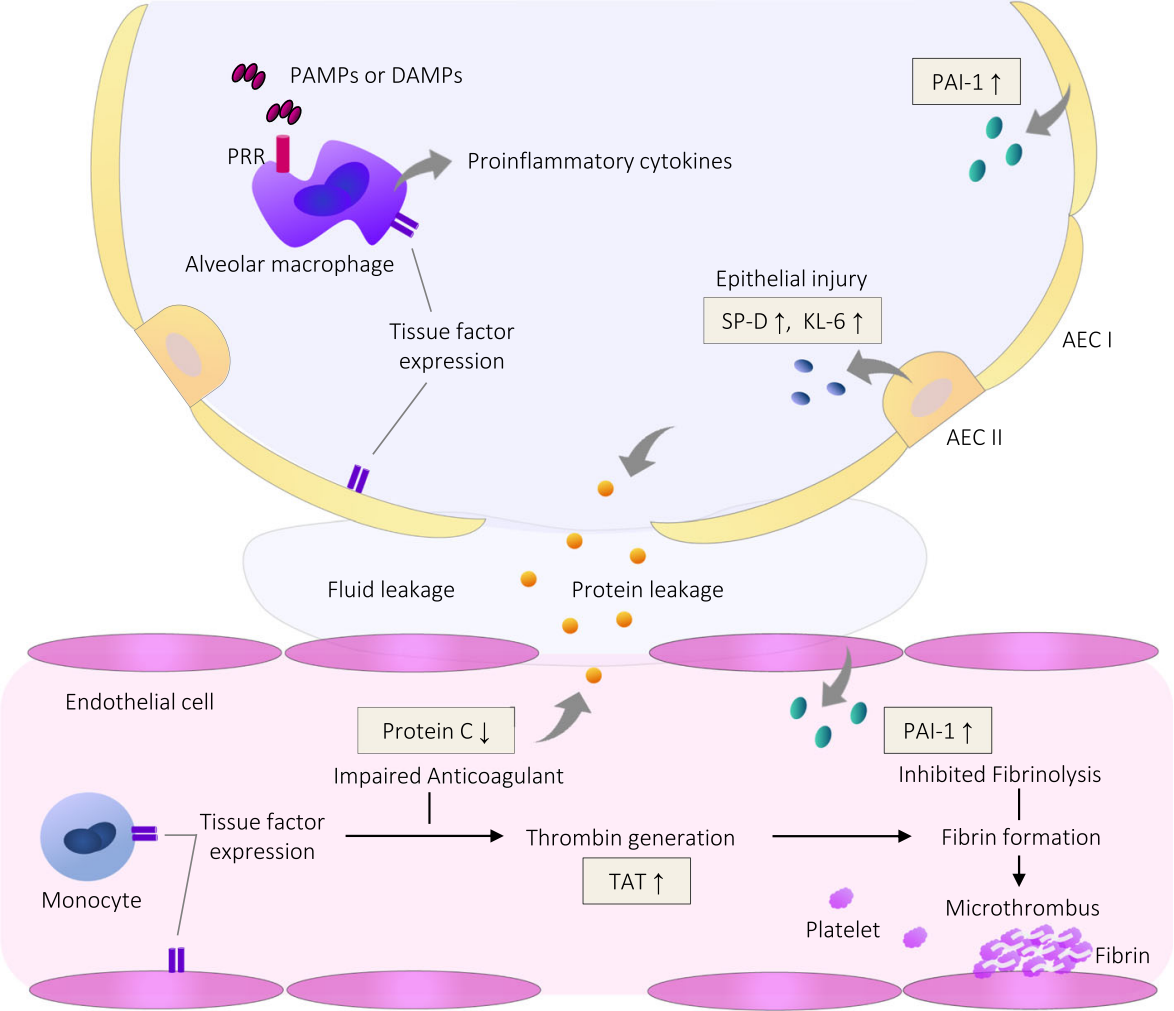
Pulmonary coagulopathy, epithelial injury, and their related biomarkers in acute respiratory distress syndrome. Proinflammatory stimuli induce the expression and production of procoagulant and antifibrinolytic factors in different cell populations and epithelial/endothelial injury in the lung. AEC, alveolar epithelial cell; DAMPS, damage-associated molecular patterns; PAI-1, plasminogen activator inhibitor-1; PAMP, pathogen-associated molecular patterns; PRR, pattern recognition receptors; SP, surfactant protein; TAT, thrombin–antithrombin complex

Activation of coagulation and alveolar epithelial injury are the hallmarks of ARDS (Fig. 1) [11, 12]. The biomarkers may reflect activation and injuries of different cell populations in the lung and thereby help to improve the understanding about pathogenic processes and to improve diagnostics. Thrombin–antithrombin complex (TAT) levels are increased in ARDS patients, reflecting tissue factor- and contact phase-mediated activation of coagulation cascade and excessive thrombin generation. Thrombin and proinflammatory cytokines activate endothelial cells, leading to expression of plasminogen activator inhibitor (PAI)-1, which inhibits fibrinolysis. The levels of natural anticoagulants such as protein C (PC) are reduced because of increased consumption, impaired synthesis, and mostly capillary leakage that results from endothelial damage. Surfactant protein (SP)-D and a membrane glycoprotein KL-6 are also increased in the plasma of ARDS patients, reflecting type II alveolar cell injury [13, 14]. The alterations in biomarkers that indicate thrombin generation, inhibited fibrinolysis, decreased anticoagulant, and epithelial injury are distinctive patterns of ARDS. However, whether these biomarker profiles may differ depending on the ARDS etiologies has not been examined.

The aim of this study was to examine the profiles of the plasma biomarkers that reflect coagulopathy and alveolar epithelial injury in patients with idiopathic/immune-related ARDS (iARDS) and in those with common direct risk factors (dARDS). We investigated the baseline levels and time courses of hemostatic and type II pneumocyte biomarkers and compared the discriminative ability of those biomarkers between iARDS and dARDS. We also evaluated the biomarkers in patients with unilateral pneumonia who were admitted during the same period for reference purposes.

## Methods

### Study design

This single-center, retrospective, observational study was conducted at a 14-bed medicosurgical intensive care unit (ICU) at Jichi Medical University Hospital (Tochigi, Japan). Medical records for all patients admitted to the ICU between April 2011 and March 2018 were reviewed. Adult patients admitted because of ARDS without indirect risk factors or unilateral pneumonia who underwent invasive mechanical ventilation within 48 h of admission were included in the study. Exclusion criteria were age < 18 years, > 1 week of respiratory disease progression before ICU admission, previously known interstitial pneumonia or IPF, or a diagnosis of *Pneumocystis* pneumonia. We also excluded patients with bone marrow failure, decompensated liver cirrhosis or failure, a history of chemotherapy, therapeutic anticoagulation, or blood transfusion during the preceding 4 weeks. The institutional research ethics committee at Jichi Medical University approved this study and waived the requirement for informed consent because of the study’s retrospective design.

### Diagnosis of pulmonary ARDS and pneumonia

The ARDS without indirect risk factors was diagnosed according to the Berlin definition with the following criteria: within 1 week of new or worsening respiratory symptoms, bilateral lung opacities were found on chest radiography, and the PaO_2_/F_I_O_2_ ratio was ≤ 300 mmHg with a positive end-expiratory pressure of ≥ 5 cmH_2_O. Additionally, no cardiac failure or fluid overload and no common indirect risk factors for ARDS, such as non-pulmonary sepsis, major trauma, or pancreatitis could be found [2]. Direct lung injury risk factors were defined as pneumonia, aspiration of gastric contents, pulmonary contusion, inhalation injury, and near drowning, based on the Berlin definitions. Patients with vasculitis were classified as having ARDS without common risk factors because vasculitis is not pathologically characterized by diffuse alveolar damage (DAD). The diagnosis of pneumonia was based on Infectious Diseases Society of America/American Thoracic Society consensus guidelines combined with clinical data and microbiological diagnostic testing (including a blood culture, sputum culture, or culture of endotracheal aspirate, and a urinary antigen test for *Streptococcus pneumoniae* and *Legionella pneumophila*) [15, 16]. Bronchoalveolar lavage (BAL) fluid for Gram staining and culture, direct fluorescence assay for *Pneumocystis jirovecii*, and a rapid influenza A/B diagnostic test (immunochromatographic assays for specific influenza viral antigens) were also performed, as needed.

ARDS without common risk factors were separated into four etiological groups, as described below [7]. Idiopathic ARDS was defined as the absence of any ARDS etiology including common risk factors despite a comprehensive diagnostic work-up, or acute presentation of idiopathic interstitial pneumonia [17]. Immune-related ARDS was defined as an acute presentation of CTD-ILD as defined in accordance with established CTD criteria (e.g., American College of Rheumatology criteria [18]) during hospitalization, or hypersensitive pneumonitis [19]. Malignancy-associated ARDS was defined as requiring cytological or pathological evidence of hematological or solid malignancy. Drug-induced ARDS was defined as previous exposure to a drug that is known to be a pneumonia inducer in the absence of any other risk factor for ARDS [20].

### Data collection

Descriptive data (including demographic, diagnostic, clinical, and laboratory data) were collected from the electronic medical records of all eligible patients. Initial severity indices, including the Acute Physiology and Chronic Health Evaluation (APACHE) II and Simplified Acute Physiology Score (SAPS) II, were calculated on the day of ICU admission [21, 22]. Sequential Organ Failure Assessment (SOFA) scores were calculated during the first 7 days [23]. Clinical outcomes were assessed according to ICU days, ventilator-free days, and all-cause 28- and 90-day mortality. For the patients with idiopathic and immune-related ARDS, BAL fluid cytological analysis and autoimmunity tests were extracted from the medical charts when available.

### Biomarker measurement

At our institute, the biomarkers of coagulation and type II pneumonocytes are routinely measured for the patients who are admitted to the ICU with respiratory failure and/or with suspected sepsis. Plasma biomarkers were measured at the time of ICU admission (ICU day 1) and on ICU days 2–5. Coagulation and fibrinolytic markers included global markers (platelet count, immature platelet fraction, prothrombin time–international normalized ratio [PT-INR], fibrin degradation product [FDP]), markers of thrombin generation (TAT), markers of anticoagulant activity (PC activity), and markers of fibrinolytic activity (plasmin–α_2_-plasmin inhibitor complex [PIC], PAI-1).

Global markers were assayed using an XE-5000 hematology analyzer (Sysmex, Kobe, Japan) and a CS-2100i automatic coagulation analyzer (Sysmex). Berichrom assays (Siemens Healthcare Diagnostics, Tokyo, Japan) were used to assay PC activity. The TAT/PIC test F enzyme immunoassay (Sysmex) was used to measure TAT and PIC levels. The PAI-1 was measured using the tPAI test (Mitsubishi Chemical Medience, Tokyo, Japan).

Surfactant protein (SP)-D, KL-6, C-reactive protein (CRP), and procalcitonin (PCT) were measured using the SP-D kit enzyme immunoassay (Yamasa, Chiba, Japan), Presto II KL-6 chemiluminescent enzyme immunoassay (Sekisui Medical, Tokyo, Japan), CRP-HG latex immunoassay (Eiken Kagaku, Tokyo, Japan), and Brahms PCT chemiluminescent enzyme immunoassay (Roche Diagnostic, Tokyo, Japan), respectively.

### Statistical analysis

Differences in clinical characteristics and laboratory data among the groups were analyzed using the *χ*^2^ test or Fisher’s exact test for categorical variables and the Wilcoxon rank-sum test or Kruskal–Wallis test with/without Steel–Dwass pairwise comparisons for continuous variables, as appropriate. Changes in the biomarker concentrations over time in the groups were compared with multiple analysis of variance. A multivariate logistic regression model based on a forward stepwise method was used to identify the best combination of coagulation biomarkers to diagnose iARDS. Receiver operating characteristic (ROC) curve analysis was performed to calculate the area under the receiver operating characteristic curve (AUC) of the biomarkers at day 1 to evaluate the discriminative capacity between the two groups. All *P* values were two-tailed, and *P* < 0.05 was considered to indicate statistical significance. Data were analyzed using JMP version 12 (SAS Institute, Tokyo, Japan).

## Results

### Characteristics of patients with iARDS, dARDS, or pneumonia

Overall, 138 ARDS patients with no indirect risk factors were admitted to the ICU during the study period. Among them, 41 were excluded based on the exclusion criteria: history of known interstitial pneumonia, 5; *Pneumocystis* pneumonia, 8; hematological malignancy with bone marrow failure, 10; liver failure, 2; anticoagulation therapy, 7; inconclusive diagnosis, 4; and insufficient data, 5. Data from the remaining 97 patients were included in the study. In addition, 39 patients who were admitted to the ICU with unilateral pneumonia during the same period were enrolled for comparison.

Among the 97 patients with pulmonary ARDS, 56 had been exposed to direct lung injury risk factors and 41 had not been exposed to any of the common risk factors. The direct risk factors of lung injury included pneumonia (42; 75.0%), aspiration (13; 23.2%), and drowning (1; 1.8%). The 41 ARDS patients without common risk factors were classified as idiopathic (17; 41.5%), immune-related (14; 34.1%), malignancy-associated (7; 17.1%), and drug-induced (3; 7.3%).

Table 1 shows the baseline characteristics and outcomes of the study patients with iARDS and dARDS and those with unilateral pneumonia. Patients with dARDS were more severely ill, with higher APACHE II, SAPS II, and SOFA scores on ICU admission compared with patients with iARDS. The PaO_2_/F_I_O_2_ ratio on admission and the severity of ARDS, however, were not different between patients with dARDS and those with iARDS. Ventilator-free days, length of ICU stay, and mortality were also similar for the two groups.

**Table 1:** Baseline characteristics and outcomes in the 124 study patients

Characteristics/outcomes	Direct-risk ARDS (*n* = 56)	Idiopathic/immune ARDS (*n* = 31)	Unilateral pneumonia (*n* = 37)	P*	P**
Demographics					
Age, years	70 (63–77.8)	66 (59–75)	66 (58.5–76)	0.19	0.39
Male, *n* (%)	41 (73.2)	16 (51.6)	25 (67.6)	*0.044*	0.13
Comorbidities, *n* (%)					
IHD	7 (12.5)	3 (9.7)	3 (8.1)	1.00	0.78
CHF	8 (14.3)	3 (9.7)	3 (8.1)	0.74	0.62
Arrhythmia	7 (12.5)	2 (6.5)	2 (5.4)	0.48	0.43
COPD	5 (8.9)	2 (6.5)	7 (18.9)	1.00	0.22
CKD	11 (19.6)	6 (19.4)	7 (18.9)	1.00	0.99
CVD	9 (16.1)	3 (9.7)	6 (13.2)	0.53	0.66
Severity of illness					
APACHE II score	31 (25–35)	23 (18–26)	24 (19–29)	*< 0.0001*	*< 0.0001*
SAPS II score	60 (52–75)	44 (27–52)	48 (35–57)	*< 0.0001*	*< 0.0001*
SOFA score					
Day 1	9 (7.3–11)	6 (5–8)	7 (6–9)	*< 0.0001*	*< 0.0001*
Maximum	11 (8–14)	7 (6–9)	8 (7.0–10.5)	*< 0.0001*	*< 0.0001*
PaO_2_/F_I_O_2_ ratio, day 1	127 (86.8–194)	119 (75–170)	192 (137–234)	0.89	*0.0035*
Set PEEP (cmH_2_O), day 1	8 (5–10)	8 (5–8)	8 (5–10)	0.58	0.86
Severity of ARDS				0.29	
Mild	13 (23.2)	4 (12.9)			
Moderate	23 (41.1)	15 (48.4)			
Severe	20 (35.7)	12 (38.7)			
Prognosis					
ICU days	11 (8–18)	10 (7–17)	10 (6.5–14)	0.82	0.45
Ventilator-free days	9.5 (0–20)	17 (0–22)	18 (14–22)	0.12	*0.021*
Mortality, *n* (%)					
28 days	12 (21.4)	4 (12.9)	1 (2.7)	0.37	*0.019*
90 days	17 (30.4)	9 (29.0)	1. (2.7)	0.89	*0.0007*

Data are expressed as the median (interquartile range) or *n* (%)

*IHD* ischemic heart disease, *CHF* chronic heart failure, *COPD* chronic obstructive pulmonary disease, *CKD* chronic kidney disease, *CVD* cerebrovascular disease, *APACHE* Acute Physiology and Chronic Health Evaluation, *SAPS* Simplified Acute Physiology Score, *SOFA* Sequential Organ Failure Assessment, *PEEP* positive end-expiratory pressure

*Comparison between patients with direct risk factor-associated ARDS and idiopathic/immune-related ARDS

**Comparison among the three groups. Italic numbers indicate statistical significance

The distribution of pathogens in patients with dARDS and those with pneumonia are shown in Additional file 1: Table S1. In patients with dARDS, the most common causative microorganisms were *Klebsiella pneumoniae* (17.9%), followed by *Streptococcus pneumoniae* (12.5%) and methicillin-susceptible *Staphylococcus aureus* (10.7%).

Among the 31 patients with iARDS, 17 (54.8%) were diagnosed with idiopathic ARDS and 14 (45.2%) with immune-related ARDS, which included the following: rheumatoid arthritis (*n* = 5), dermatomyositis (*n* = 3), systemic lupus erythematosus (*n* = 2), scleroderma (*n* = 1), microscopic polyangiitis (*n* = 1), granulomatosis with polyangiitis (*n* = 1), and hypersensitivity pneumonitis (*n* = 1). Table 2 shows the BAL findings and autoantibodies in patients with iARDS. In about half of these patients (idiopathic, 62.5%; immune-related, 42.9%), neutrophils and lymphocytes were both elevated in BAL fluid, showing a mixed cellular pattern (defined as neutrophil > 3% and lymphocyte > 15% on BAL differential cell counts). Antinuclear antibody was positive (with > 1:160 titers) in 64.3%, and anticyclic citrullinated peptide antibody was positive in 35.7% of the patients with immune-related ARDS. Notably, 23.5% of the patients with idiopathic ARDS were positive for autoantibodies against aminoacyl-tRNA synthetase.

**Table 2:** Bronchoalveolar lavage and autoantibody results in patients with iARDS

Parameter	Idiopathic ARDS (*n* = 17)	Immune-related ARDS (*n* = 14)
BAL, *n* (%)	8 (47.1)	7 (50.0)
Cell count, 10^5^/mL	7.5 (4.1–13.3)	12.0 (3.7–17.3)
Cell types (%)		
Macrophages	36.3 (25.2–57.8)	31.3 (18.0–49.0)
Neutrophils	27.2 (5.7–58.5)	41.0 (5.2–77.0)
Lymphocytes	21.7 (10.1–40.8)	24.5 (2.5–51.4)
Hemorrhage	1 (12.5)	4 (57.1)
Neutrophilic pattern	2 (25.0)	3 (42.9)
Lymphocytic pattern	1 (12.5)	1 (14.3)
Mixed pattern	5 (62.5)	3 (42.9)
Autoantibodies, *n* (%)		
ANA	3 (17.4)	9 (64.3)
CCP	2 (11.8)	5 (35.7)
ANCA	0	2 (14.3)
SSc-associated	0	2 (14.3)
RNP	0	1 (7.1)
ARS	4 (23.5)	0

*BAL* bronchoalveolar lavage, *ANA* antinuclear antibody, *CCP* cyclic citrullinated peptide, *ANCA* anti-neutrophil cytoplasmic antibody, *SSc* systemic sclerosis, *RNP* ribonucleoprotein, *ARS* aminoacyl-tRNA synthetases

### Coagulation biomarkers in patients with iARDS or dARDS

**Fig. 2:**
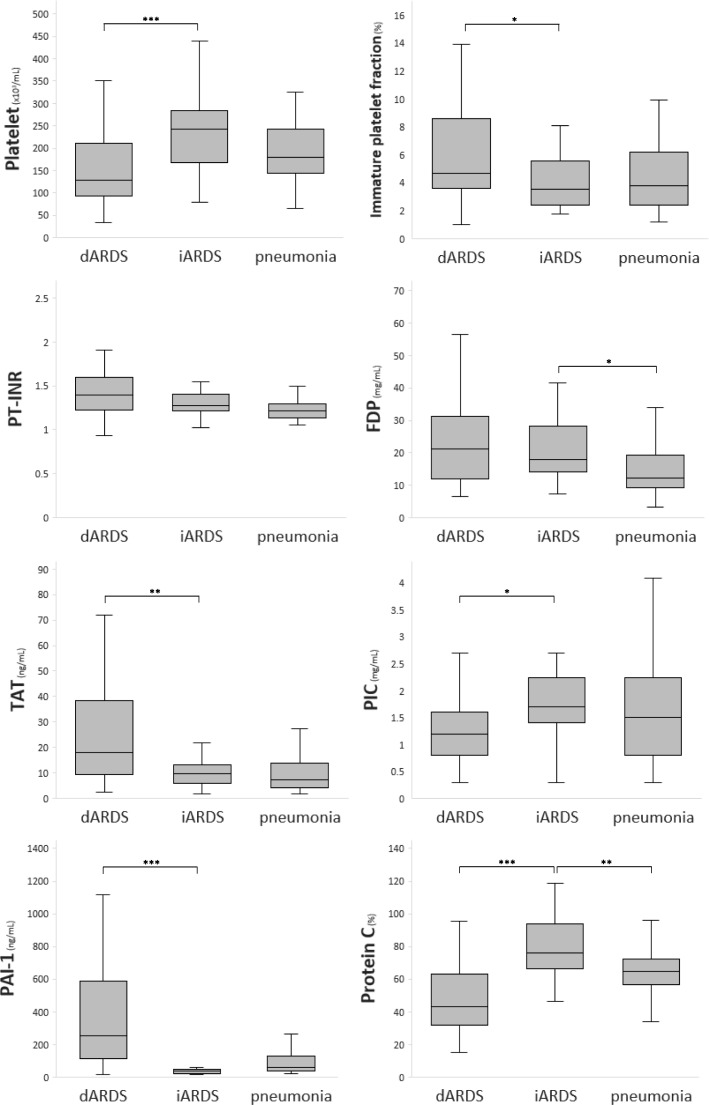
Coagulation biomarkers on the day of admission in patients with iARDS, dARDS, or pneumonia. Box plot shows the median levels of biomarkers with interquartile ranges. The whiskers denote the maximum and minimum values within the 1.5 times interquartile ranges. PT-INR, prothrombin time–international normalized ratio; FDP, fibrin degradation products; TAT, thrombin–antithrombin complex; PIC, plasmin–α_2_-plasmin inhibitor complex; PAI-1, plasminogen activator inhibitor-1. Steel–Dwass test was used for multiple pairwise comparisons. *P < 0.05, **P < 0.01, ***P < 0.001

**Fig. 3:**
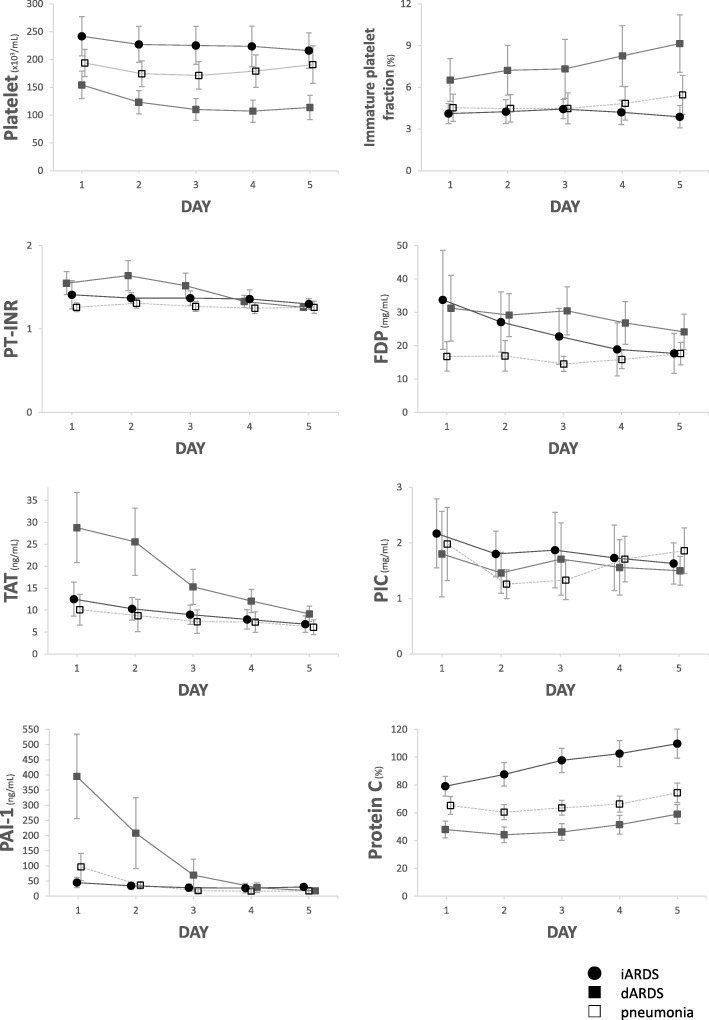
Changes in coagulation biomarkers during days 1–5 in the intensive care unit (ICU) for patients with iARDS, dARDS, or pneumonia. PT-INR, prothrombin time–international normalized ratio; FDP, fibrin degradation products; TAT, thrombin–antithrombin complex; PIC, plasmin–α_2_-plasmin inhibitor complex; PAI-1, plasminogen activator inhibitor-1. Data are expressed as the mean, with the 95% confidence interval shown by the error bars

Figure 2 shows the results of multiple comparisons for coagulation biomarkers on day 1 in patients with iARDS, dARDS, and unilateral pneumonia. The changes in coagulation biomarkers over time among the three groups are shown in Fig. 3. In the iARDS and dARDS patients, the PT-INR and FDP were increased to similar levels on day 1: INR 1.28 (1.21–1.42) vs. 1.40 (1.22–1.60), *P* = 0.19; FDP 18.0 (13.7–29.7) vs. 21.2 (11.7–31.6) mg/mL, *P* = 0.99, respectively (Fig. 2), and during the observational period (overall difference: INR, *P* = 0.41; FDP, *P* = 0.36; Fig. 3), suggesting a hypercoagulable state in both groups. The TAT levels were increased in the three groups, but those levels were much lower in iARDS patients compared with dARDS patients on day 1 (9.8 [5.9–13.5] vs. 18.2 [9.4–40.5] ng/mL, *P* = 0.0046) and during the observational period (overall difference, *P* = 0.0029), and they were similar to those in patients with unilateral pneumonia. PAI-1 levels were significantly higher (251 [95.8–629] vs. 38.0 [17.5–51.0] ng/mL, *P* < 0.0001) and the PIC levels significantly lower (1.2 [0.75–1.7] vs. 1.7 [1.3–2.3] mg/mL, *P* = 0.014) on day 1 in patients with dARDS than in those with iARDS, suggesting inhibited fibrinolysis in the dARDS patients. Further, PC activity (marker of the anticoagulant system) was significantly lower in patients with dARDS compared with those with iARDS (42.9 [30.9–63.6] vs. 76.2 [66.1–95.2] %, *P* < 0.0001). When we compared the PC activities in patients with iARDS and unilateral pneumonia, those values were still higher in patients with iARDS. Among the coagulation biomarkers, a multivariate stepwise logistic regression analysis revealed that PAI-1 and PC activity comprised the best combination with which to identify patients with iARDS.

### Biomarkers for infection and pneumocytes in patients with iARDS or dARDS

**Fig. 4:**
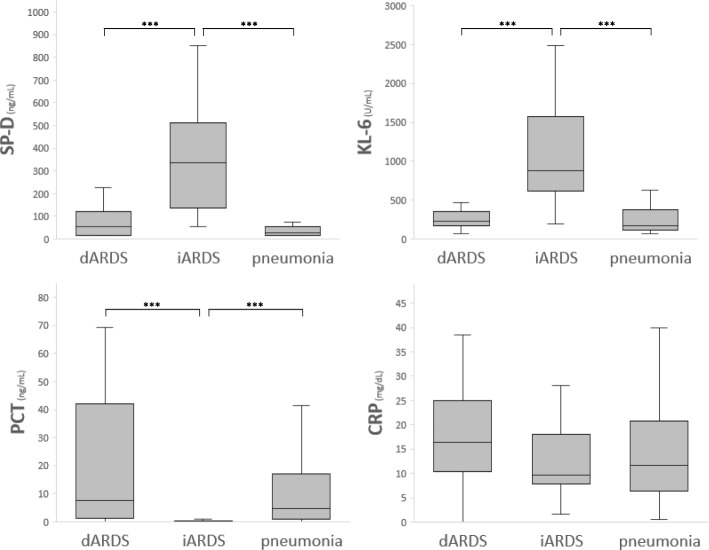
Biomarkers of infection or epithelial injury on the day of admission for patients with iARDS, dARDS, or pneumonia. Box plot shows the median levels of biomarkers with interquartile ranges. The whiskers denote the maximum and minimum values within the 1.5 times interquartile ranges. PCT, procalcitonin; CRP, C-reactive protein; SP, surfactant protein. The Steel–Dwass test was used for multiple pairwise comparisons. ***P < 0.001

**Fig. 5:**
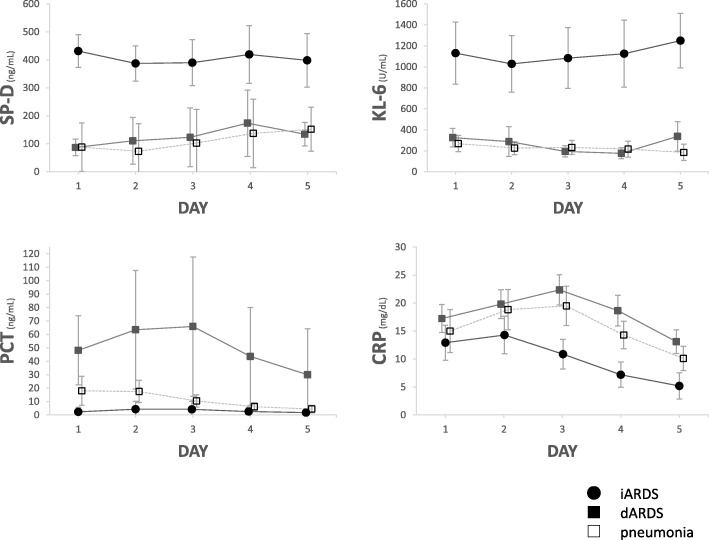
Changes in plasma biomarkers of infection or epithelial injury during days 1–5 in the ICU for patients with iARDS, dARDS, or pneumonia. PCT, procalcitonin; CRP, C-reactive protein; SP, surfactant protein. Data are expressed as the mean, with the 95% confidence interval shown by the error bars

PCT levels (marker of infection) on day 1 were increased in the dARDS and pneumonia patients but were lower than the reference value for infection in patients with iARDS. However, levels of CRP, a widely used marker of inflammation and mechanistically downstream of IL-6, were not different among the three groups. The markers of type II pneumocyte injury, SP-D, and KL-6 were markedly increased in patients with iARDS compared with those with dARDS or pneumonia (Figs. 4 and 5).

### Capacity of plasma biomarkers to distinguish between ARDS subtypes at baseline

To compare the abilities of the plasma biomarkers to distinguish between ARDS subtypes, we conducted a ROC curve analysis to calculate the AUCs of biomarkers for coagulation, infection, and pneumocytes (Table 3). We found that the AUCs for discriminating between iARDS and dARDS were high (> 0.8) for PAI-1, PC activity, PCT, SP-D, and KL-6. These AUC results showed that the ability to distinguish between iARDS and dARDS was comparable among those five biomarkers (PC, 0.86 [0.76–0.93], *P* = 0.33; PAI-1, 0.89 [0.74–0.96], *P* = 0.95; PCT, 0.89 [0.79–0.96], *P* = 0.66; and SP-D, 0.88 [0.77–0.94], *P* = 0.16; vs. KL-6, 0.90 [0.79–0.95], respectively).

**Table 3:** Area under the ROC curve of plasma biomarkers for distinguishing between iARDS and dARDS

Biomarkers	AUC	95% CI
Coagulation		
Platelet count	0.77	0.65–0.86
Immature platelet fraction	0.69	0.54–0.81
PT-INR	0.61	0.49–0.73
FDP	0.51	0.38–0.63
TAT	0.72	0.59–0.82
PIC	0.69	0.56–0.80
PAI-1	0.89	0.74–0.96
Protein C	0.86	0.76–0.93
Infection/inflammation		
PCT	0.89	0.79–0.96
CRP	0.65	0.52–0.76
Alveolar epithelial injury		
SP-D	0.88	0.77–0.94
KL-6	0.90	0.79–0.95

*AUC* area under the receiver characteristics curve, *CI* confidence interval, *PT-INR* prothrombin time–international normalized ratio, *FDP* fibrin degradation products, TAT thrombin–antithrombin complex, PIC plasmin–α_2_-plasmin inhibitor complex, *PAI-1* plasminogen activator inhibitor-1, *PCT* procalcitonin, *CRP* C-reactive protein, *SP* surfactant protein

## Discussion

In this retrospective analysis of ARDS subtypes, we evaluated the changes in coagulation and alveolar epithelial cell biomarkers over time in patients with iARDS and dARDS. TAT and PAI-1 levels were increased in patients in both ARDS subgroups, but a significantly higher increase in those biomarkers were observed in patients with dARDS. Additionally, PC activity decreased in dARDS, whereas that in iARDS was normal or even increased. There were also significant differences in PCT, SP-D, and KL-6 levels between the two groups on the day of ICU admission. These results suggest that each iARDS and dARDS may have its distinct patterns of plasma biomarkers, which could help to differentiate between these ARDS subgroups.

Alterations in coagulation and fibrinolytic abnormalities have been observed in animal models of lung injury and in human patients with ARDS or ILD [24–26]. Chambers reported that uncontrolled activation of the coagulation cascade might contribute to the development of fibrosis in both ARDS and IPF, suggesting that coagulopathy is pivotal as a common pathophysiological factor in these diseases [27]. In our study, increased coagulation (suggested by increased TAT) and suppressed fibrinolysis (suggested by elevated PAI-1 levels) were observed in patients with dARDS but were less prominent in iARDS patients. These results are in line with Gunther et al.’s study that showed enhanced procoagulant and depressed fibrinolytic capacities were greater in patients with ARDS than in those with pneumonia or in healthy controls [28, 29]. In addition, there were significant differences in coagulation inhibition or the levels of natural anticoagulant between dARDS and iARDS. To the best of our knowledge, this is the first study to show differences in the coagulation profile between ARDS with and without common risk factors, or ARDS mimics.

The pathophysiology accounting for these different coagulopathic patterns has not been identified. One explanation might be that inflammation and coagulopathy are relatively limited to the lung in iARDS, whereas dARDS is a more systemic disease. Although the cause of dARDS is direct lung injury, indicators of systemic involvement, reflected in the APACHE II or SOFA scores, were significantly higher in patients with dARDS compared with those with iARDS. Another possible mechanism might be explained by the different pathological findings of iARDS and dARDS. Lorente et al. showed that ARDS patients with DAD had higher PT-INR and lower platelet counts than ARDS patients without DAD [30].

PC activities were within the normal range or even increased in iARDS patients, whereas those in dARDS patients remained significantly decreased throughout the observational period. These results are somewhat consistent with the meta-analysis conducted by Terpstra et al., which showed that the PC level was decreased in ARDS and was associated with increased odds for an ARDS diagnosis [14]. In the presence of sepsis or ARDS, anticoagulation pathways, such as the PC system, are impaired because of increased consumption, decreased protein synthesis, extravasation from vessels, and degradation by several proteolytic enzymes. Particularly, extravascular leakage resulting from endothelial damage may be the main mechanism during the acute phase [31, 32]. Decreased PC activity in dARDS patients, therefore, may reflect systemic endothelial dysfunction. In contrast, Bargagli et al. reported that PC activity increased during acute exacerbation of usual IP but was normal in stable usual IP or NSIP [33]. They postulated that increased PC activity was associated with upregulation of the fibrinolytic response to a procoagulant state caused by fibrosis. Although the pathophysiological mechanisms of altered PC activity in patients with ARDS have not been clarified, our findings indicate that the differences in the anticoagulant response to increased coagulation may be useful for distinguishing the ARDS etiologies.

We analyzed idiopathic and immune-related ARDS within the same category, although these two disorders are classified as having different etiologies. Idiopathic interstitial pneumonias (IIPs) are diffuse inflammatory lung diseases that are grouped together with similar clinical, radiological, and histopathological features. The diagnosis of an IIP is based on the exclusion of known causes of IP, such as drugs, environmental exposure, or CTDs [17]. CTD-ILDs are the lung manifestation of CTDs, where the underlying mechanism is systemic autoimmunity. Thus, the diagnosis is based on specific extra-thoracic features of CTDs with/without the existence of autoantibodies. Recent studies have shown, however, that some patients with ILD have certain clinical features that suggest an underlying autoimmune process, although they do not fully meet the diagnostic criteria for any characterizable CTD. The European Respiratory Society/American Thoracic Society Task Force on Undifferentiated Forms of Connective Tissue Disease-Associated Interstitial Lung Disease proposed the term “interstitial pneumonia with autoimmune features” for such diseases [9]. In our study, approximately 20% of the idiopathic ARDS patients were diagnosed as having anti-synthetase syndrome without myositis or arthritis and 10% were positive for anticyclic citrullinated peptide antibody. The biomarker profiles were similar in patients with idiopathic ARDS and those with immune-related ARDS, which indicates overlapping pathophysiology of coagulopathy and epithelial injury in these two subsets.

SP-D and KL-6, which are glycoproteins secreted by type II alveolar epithelial cells, are widely used as potential surrogate markers of alveolar injury, or alveolitis. The roles of SP-D and KL-6 are well established for improving diagnostic accuracy, predicting the prognosis, or predicting the risk of acute exacerbation, especially in patients with NSIP or IPF [13, 34, 35]. SP-D and KL-6 are also known to be elevated in ARDS patients [14, 36], but no published reports have compared the biomarker levels according to different ARDS etiologies. Using data from Korea and the USA, Park et al. showed that plasma SP-D levels were elevated (median, 20.8 ng/mL; interquartile range, 12.7–38.4 ng/mL) in patients with ARDS mostly due to pneumonia (87.2% of the study population) [37]. Ohnishi et al. compared SP-D and KL-6 levels in patients with ILD (including IPF and CVD-associated interstitial pneumonia) with those in patients with bacterial pneumonia and healthy subjects [13]. They identified the cutoff values for the diagnosis of ILDs as 116 ng/mL for SP-D and 465 U/ mL for KL-6. In our study, SP-D and KL-6 levels were significantly higher in patients with iARDS compared with those with dARDS (SP-D 336 [134–538] vs. 54.9 [17.2–123] ng/mL; KL-6884 [577–1680] vs. 228 [161–363] U/mL). Our results were consistent with those in previous studies and provided new evidence that the elevated levels of biomarkers for alveolar epithelial injury may differ depending on the ARDS subtype.

### Limitations

There were some potential limitations to our study. First, this was a retrospective, observational study conducted at a single center with a relatively small population. A large validation study is needed to confirm our results. Second, we could not perform serological tests for non-influenza respiratory viruses, such as the respiratory syncytial virus or human metapneumovirus. Although we ruled out the common ARDS risk factors and known causes of interstitial pneumonia (e.g., drugs, environmental agents, CTDs) to diagnose idiopathic ARDS, we could not completely exclude the possibility of viral infections or environmental antigen exposures, which could subside spontaneously. Third, we could perform BAL for only about half of iARDS patients, which may not be generalizable to the whole population. Finally, we did not measure the biomarkers in the BAL fluid. Although systemic markers are easier to obtain and the BAL procedure may not always be possible because of the risk of respiratory and hemodynamic complications, the biomarkers in the BAL fluid would more specifically reflect the regional pathophysiology in the alveoli. Further studies are needed to evaluate the pathogenic processes of these biomarkers from the pulmonary compartment to the circulation.

## Conclusions

Changes in the biomarkers of coagulopathy and alveolar epithelial injury were observed in both patients with dARDS and with iARDS, but those biomarker profiles were significantly different between the two groups. PAI-1 and PC activity, as well as PCT, SP-D, and KL-6, discriminated well between dARDS and iARDS on the day of ICU admission. These preliminary findings indicate that the biomarker profiles may help to understand the pathogenic processes and improve the prompt differentiation between ARDS subtypes.

## Additional file

Additional file 1: **Table S1.** Distribution of microorganisms in patients with dARDS or pneumonia. (DOCX 23 kb)

## Data Availability

The dataset generated and/or analyzed during the current study is not publicly available because of patient-related confidentiality, but is available from the corresponding author upon reasonable request.
