# Supplementary material for: Biomarker profiles of coagulopathy and alveolar epithelial injury in acute respiratory distress syndrome with idiopathic/immune-related disease or common direct risk factors
Source: Crit Care. 2019 Aug 19;23:283. doi: 10.1186/s13054-019-2559-6 (PMC6699073; doi:10.1186/s13054-019-2559-6)
Supplement: Supplementary file 1 — Table S1. Distribution of microorganisms in patients with dARDS or pneumonia. (DOCX 23 kb) [file 13054_2019_2559_MOESM1_ESM.docx]

**Table S1.** Distribution of microorganisms in patients with dARDS or pneumonia

| Microorganisms | dARDS  (n=56) | Unilateral pneumonia  (n=37) |
| --- | --- | --- |
| *Klebsiella pneumoniae* | 10 (17.9%) | 3 (8.1%) |
| *Streptococcus pneumoniae* | 7 (12.5%) | 7 (18.9%) |
| *Staphylococcus aureus* |  |  |
| Methicillin-susceptible | 6 (10.7%) | 4 (10.8%) |
| Methicillin-resistant | 5 (8.9%) | 4 (10.8%) |
| *Haemophilus influenzae* | 3 (5.4%) | 2 (5.4%) |
| *Pseudomonas aeruginosa* | 1 (1.8%) | 5 (13.5%) |
| *Escherichia coli* | 1 (1.8%) | 1 (2.7%) |
| *Enterobacter aerogenes* | 1 (1.8%) | – |
| *Moraxella catarrhalis* | 1 (1.8%) | – |
| *Acinetobacter* sp. | – | 1 (2.7%) |
| *Bacteroides* sp. | 1 (1.8%) | – |
| *Legionella pneumophila* | 1 (1.8%) | 2 (5.4%) |
| *Chlamydia pneumoniae* | 1 (1.8%) | – |
| *Mycoplasma pneumoniae* | – | 1 (2.7%) |
| *Influenza* virus | 6 (10.7%) | 3 (8.1%) |

dARDS, direct common risk factor-related acute respiratory distress syndrome
